# Structure, Function, and Therapeutic Potential of Marine Bioactive Peptides

**DOI:** 10.3390/md17090505

**Published:** 2019-08-28

**Authors:** Tatiana V. Ovchinnikova

**Affiliations:** M.M. Shemyakin & Yu.A. Ovchinnikov Institute of Bioorganic Chemistry, the Russian Academy of Sciences, Miklukho-Maklaya str. 16/10, 117997 Moscow, Russia; ovch@ibch.ru; Tel.: +7-495-336-44-44

In recent years, bioactive peptides from marine organisms have gained increasing attention in the field of pharmaceutical, cosmeceutical, and nutraceutical product development owing to their interesting biological properties. They are involved in fundamental mechanisms that allow the survival of living organisms, including their defense, reproduction, growth, and homeostasis. Marine peptides that are diverse in structure and function have been found in various phyla, and their number has dynamically grown over the recent years. Some of them are evolutionary ancient molecular factors of innate immunity that play a key role in host defense [1]. Long-term evolution of marine organisms proceeded in continuous contact with pathogens, and efficient defense mechanisms were the necessary condition of their survival. Peptides with protective functions were isolated from tissues of many marine invertebrates and vertebrates. A plethora of biological activities, including antibacterial, antifungal, antiviral, cytotoxic, neurotoxic, anticoagulant, antidiabetic, antifreeze, endotoxin-binding, and immune-modulating, make marine peptides an attractive molecular basis for the design of innovative antibiotics, anticancer drugs, analgetics, medicines for neurological disorders, etc.

The Special Issue “Marine Bioactive Peptides: Structure, Function, and Therapeutic Potential” was aimed at collecting papers on up-to-date information regarding isolation, structural elucidation, functional characterization, and evaluation of the therapeutic potential of peptides from marine organisms. Chemical synthesis and biotechnological production of marine peptides and their mimetics were also a focus of this Special Issue. In total, 24 papers were accepted and included in the Special Issue, which are now published as a book. Getting started with this book, we planned to produce an interesting edition that would cover breakthroughs and recent trends in basic and applied research on marine peptides. 

Bioactive peptides showing antihypertensive, antioxidative, and antidiabetic activities were isolated from seaweed [2]. Algae peptides have strong potential for use as therapeutic drugs, especially for treatment of cardiovascular diseases and diabetes, and as functional food formulations in health care. This Special Issue includes papers analyzing potential health impacts of bioactive peptides from the seaweed species *Pyropia yezoensis*, *Chlorella pyrenoidosa*, and *Gracilariopsis lemaneiformis*. In particular, the protective effects of the *P. yezoensis* peptide PYP15 against dexamethasone-induced myotube atrophy was revealed [3]. The effect of the *Chlorella pyrenoidosa* protein hydrolysate (CPPH) and *Chlorella pyrenoidosa* protein hydrolysate-calcium chelate (CPPH-Ca) on calcium absorption and gut microbiota composition was evaluated [4]. It was demonstrated that CPPH-Ca could promote calcium absorption partially through regulating specific gut microbiota and modulating expressions of the calcium absorption-related genes in kidney. Therefore, CPPH-Ca may be used to promote the calcium absorption and reduce the risk of calcium deficiency [4]. Two novel angiotensin-converting enzyme (ACE) inhibitory peptides were identified in the *Gracilariopsis lemaneiformis* protein hydrolysate. Both peptides were noncompetitive inhibitors of ACE and reduced systolic and diastolic blood pressure in spontaneously hypertensive rats [5]. 

All members of the phylum Cnidaria (sea anemones, corals, jellyfish, and hydra) are venomous [6]. Acid-sensing ion channel 3 (ASIC3) makes an important contribution to development and maintenance of inflammatory and acid-induced pain. An inhibition of ASIC3 was reported as an attractive approach to inducing analgesia. Different ASIC3 inhibitors including the peptides APETx2 and Ugr9-1 from the venom of the sea anemone *Urticina grebelnyi* and nonpeptide molecules sevanol and diclofenac were compared with a focus on their anti-inflammatory and analgesic effects [7]. All the tested compounds had distinct effects on pH-induced ASIC3 current. Comparison of the ASIC3-selective ligands in different animal pain models provides an opportunity to estimate their pharmacological potential and specify the properties of the most attractive compound for analgetics development [7]. The antitumor effect of the sea anemone *Anthopleura anjunae* peptide AAP-H in prostate cancer DU-145 cells was investigated in vitro and in vivo [8]. The obtained results indicated that AAP-H was nontoxic and exhibited the antitumor activity. The role of the phosphatidylinositol 3-kinase/protein kinase B/mammalian rapamycin target protein (PI3K/AKT/mTOR) signaling pathway in the antitumor mechanism of APP-H was investigated. It was shown that the antitumor mechanism of APP-H on DU-145 cells may involve regulation of the PI3K/AKT/mTOR signaling pathway, which eventually promotes apoptosis via mitochondrial and death receptor pathways [8]. 

Echinoderms are ancient marine invertebrates. The phylum Echinodermata contains about 7000 living species including sea stars (asteroids), sea urchins (echinoids), brittle stars (ophiuroids), sea lilies (crinoids), and sea cucumbers (holothurians). Previously, natural ACE-inhibitory peptides were obtained from the sea cucumber protein hydrolysates by a plastein reaction [9,10]. The aim of the published work was to prepare efficient ACE-inhibitory peptides from sea cucumber-modified hydrolysates by adding exogenous amino acids. Two novel efficient ACE-inhibitory peptides were purified and identified from the sea cucumber *Acaudina molpadioidea* which may be useful in the preparation of antihypertensive drugs [11]. 

Synthesis of bioactive peptides was detected in the neuroendocrine, immune, and gut system of mollusks [12]. A novel peptide, isolated from the abalone *Haliotis discus hannai* and designated as AATP, effectively inhibited matrix metalloproteinases (MMPs) by blocking MAPKs and NF-κB pathways, leading to the downregulation of metastasis of tumor cells [13]. Moreover, AATP significantly inhibited vasculogenic mimicry and pro-angiogenic factors, including vascular endothelial growth factor and MMPs by suppression of AKT/mTOR signaling. The anti-metastatic and anti-vascular effect of AATP in HT1080 cells revealed that AATP may be a potential anticancer lead compound [13]. Zinc-binding peptides were prepared from the oyster *Crassostrea gigas*’ hydrolysates modified by the plastein reaction, and the zinc absorption mechanism of the peptide-zinc complex was examined [14]. The complex was shown to promote the intestinal absorption of zinc and have a great potential for zinc supplementation as a functional food ingredient [14]. Two novel multi-functional peptides were isolated from the marine snail *Neptunea arthritica cumingii*. Both peptides showed antioxidant, antidiabetic, and ACE-inhibitory activities [15]. The venom of each *Conus* species consists of a diverse array of neurophysiologically active peptides. Isolation and characterization of the first bioactive peptide from the venom of *Conus ateralbus*, named conotoxin AtVIA, was described [16]. AtVIA manifested an excitatory activity in mouse lumbar dorsal root ganglion neurons. AtVIA has homology with δ-conotoxins from other worm-hunters, including conserved elements in amino acid sequences of δ-conotoxins from fish-hunting *Conus*. The presence of δ-conotoxins that act on vertebrate Na^+^-channels has thus been established in two divergent worm-hunting clades. The results are consistent with the hypothesis that certain worm-hunting *Conus* evolved δ-conotoxins that act to probably deter competitors in a defensive envenomation strategy [16]. To analyze putative conotoxin transcripts from the venom ducts of three vermivorous cone snails (*Conus caracteristicus*, *Conus generalis*, and *Conus quercinus*), high-throughput transcriptome sequencing was performed [17]. In total, 118, 61, and 48 putative conotoxins (across 22 superfamilies) were identified from the three *Conus* species, respectively [17]. Two comprehensive reviews included in this Special Issue deal with structural and functional analyses of cone snail toxins [18] and with computational studies on the conopeptides [19], providing a good overview of bioactive peptides from venoms of *Conus* species and their therapeutic potential.

Polychaeta is an almost uninvestigated class of invertebrates in the context of discovery of new host defense peptides. The large majority of polychaeta species are marine animals that inhabit all oceans and seas from the Arctic to the Antarctic. On the grounds of their morphology and physiology, polychaeta are considered as the most primitive annelids [20]. Papers included in this Special Issue deal with marine polychaeta, providing good examples of their biological potential. The peptides arenicins and nicomicins, isolated from *Arenicola marina* [21,22] and *Nicomache minor* [23], respectively, exhibited in vitro antimicrobial activity and possessed cytotoxicity against cancer cells. Arenicin was shown to modulate the human complement system [22]. At relatively low concentrations, the peptide stimulates complement activation and lysis of target erythrocytes, whereas at higher concentrations arenicin acts as a complement inhibitor [22]. The peptide PAP with anticancer activity was purified from the enzymatic hydrolysate of *Perinereis aibuhitensis* [24]. PAP inhibited proliferation and induced apoptosis of human lung cancer H1299 cells. The peptide may be used for prevention or treatment of human non-small cell lung cancer [24]. 

Crustaceans form a diverse arthropod taxon which contains about 67,000 described species including crabs, shrimps, lobsters, crayfish, prawns, krill, woodlice, and barnacles. Many crustaceans are free-living marine animals. A novel antimicrobial peptide polyphemusin III from the horseshoe crab *Limulus polyphemus* was examined against bacterial strains and human cancer, transformed, and normal cell cultures [25]. The peptide showed cytotoxic activity and caused fast permeabilization of the cytoplasmic membrane of human leukemia cells HL-60. In comparison to known polyphemusins and tachyplesins, polyphemusin III demonstrated a similar or lower antimicrobial effect, but significantly higher cytotoxicity against human cancer and transformed cells [25]. Anti-lipopolysaccharide factors (ALFs) are β-hairpin peptides with the ability to bind to microbial surface molecules. ALFs appear to be exclusive to marine chelicerates and crustaceans. The remarkable diversity of ALFs was demonstrated in the *Litopenaeus vannamei* shrimp [26]. At least seven members of the ALF family were found, all of which were encoded by different loci with conserved gene organization. The transcriptional profile of ALFs was compared in terms of tissue distribution, response to pathogens and shrimp development. ALFs were found to be constitutively expressed in hemocytes and to respond differently to tissue damage. ALFs form a family of shrimp peptides that has been the subject of intense diversification. These data suggest that multiple selection pressures have led to functional diversification of ALFs in shrimp [26]. 

Fish-derived bioactive peptides suggested to influence pathways involved in regulation of blood pressure, lipid and glucose metabolism, and body composition. Some of them could be developed as antihypertensive components in functional foods or nutraceuticals. The protective effects of the tilapia *Oreochromis niloticus* peptide against oxidative stress, inflammation, and endothelial injury were evaluated in angiotensin II (Ang II)-stimulated human umbilical vein endothelial cells [27]. The peptide moderated Ang II-stimulated oxidative stress and vascular endothelial dysfunction [27]. Another tilapia peptide with a high ACE-inhibitory activity was identified, and its antihypertensive effect was evaluated in vivo [28]. The obtained results showed that the tilapia peptide exerts an antihypertensive effect in spontaneously hypertensive rats, and the systolic and diastolic blood pressures of the rats remarkably decreased [28]. Thus, both tilapia peptides have potential for application in therapy of hypertensive disorders. Eight antioxidant peptides were purified from the hairtail *Trichiurus japonicas* [29]. The peptides might serve as potential antioxidants in pharmaceutical and health food industries. Several novel short antibacterial peptides were isolated from the half-fin anchovy *Setipinna taty* [30]. The peptides displayed antibacterial activity against *Escherichia coli* via inducing intracellular H_2_O_2_ production [30]. A novel peptide inhibiting the influenza A H1N1 virus neuraminidase was isolated from the skin hydrolysates of the cod *Gadus macrocephalus* [31]. The peptide acts as a neuraminidase blocker inhibiting influenza A virus in Madin–Darby canine kidney (MDCK) cells. Thus, the peptide has potential utility in treatment of the influenza virus infection.

As seen from the above overview, the papers included in this Special Issue deal with diverse marine organisms, providing an overall view of their biological potential. A range of new marine-derived peptides were isolated from evolutionarily distant species and characterized. Significantly, most of them displayed broad-spectrum biological activities and potential for use in clinical trials in humans. All the papers presented in this Special Issue underline the central role of bioactive peptides in innate immunity of marine organisms as well as their therapeutic potential for human health care. 

In conclusion, the Guest Editor thanks all the authors who contributed to this Special Issue, all the reviewers for evaluating the submitted manuscripts, and the Editorial board of *Marine Drugs*, especially Orazio Taglialatela-Scafat, Editor-in-Chief of this journal, and Estelle Fan, Assistant Editor, for their kind help in bringing this book into reality.
